# Three-year functional outcome after open pelvic fracture treatment: a retrospective case series from a level I trauma center

**DOI:** 10.1007/s00590-022-03234-x

**Published:** 2022-02-28

**Authors:** Yi-Hsun Yu, Yung-Heng Hsu, Ying-Chao Chou, Chang-Heng Liu, I.-Chuan Tseng, I.-Jung Chen

**Affiliations:** Department of Orthopedic Surgery, Musculoskeletal Research Center, Chang Gung Memorial Hospital and Chang Gung University, 5 Fu-Hsin Street, Kweishan, Tao-Yuan, 33302 Taiwan

**Keywords:** Open pelvic fracture, Functional outcome, Retrospective, Treatment protocol

## Abstract

**Purpose:**

Open pelvic fractures have high mortality rates, and survivors may have ongoing functional deficits from severe trauma and invasive life-saving procedures. However, there are limited reports regarding the functional status evaluation following open pelvic fractures. We aimed to report the treatment experiences and short-term functional outcomes of patients with open pelvic fractures.

**Methods:**

We retrospectively reviewed the data of 19 consecutive patients with pelvic fractures who underwent treatment at a single institute between January 2014 and June 2018. The resuscitation protocol, osteosynthesis strategy, reduction quality of the pelvic ring, and functional outcomes were analyzed.

**Results:**

The incidence and mortality rates in patients with open pelvic fractures were 4.9 and 21.6%, respectively. Ten, one, and seven of the open wounds related to the pelvic fractures were located in Faringer zones I, II, and III, respectively. Fractures of four patients were categorized as classes 1 and 2, and those of 11 patients as class 3, according to the Jones–Powell classification. Eleven of 19 (57.9%) and 9 of 19 (47.5%) revealed excellent reduction quality by Matta/Torenetta and Lefaivre criteria, respectively. The Merle d'Aubigné score improved at each evaluation but stagnated after 24 months. The Majeed hip score also improved at the 12-month evaluation but the improvement stopped thereafter. At a 3-year follow-up, the patients with excellent reduction of the pelvic ring showed the highest functional performances.

**Conclusion:**

Improvements in functional status of patients with open pelvic fractures can be anticipated based on the reduction quality of the pelvis ring.

## Introduction

Many pelvic fractures occur during high-energy activities. The pelvic fracture itself, or the concomitant injuries, may lead to life-threatening conditions and further long-term or permanent functional deficits [1–3]. Therefore, the timely and optimal management of such injuries is a challenge for orthopedic surgeons [4, 5].

With advancements in resuscitation procedures and improvements in the understanding of surgical anatomy as well as in surgical skills, a functional status following closed pelvic fractures comparable to the pre-injury status may be achieved [6, 7]. However, major organ injuries in patients with open pelvic fractures may be systemic, thus making the functional recovery unpredictable. There are limited reports regarding the functional status evaluation following open pelvic fractures in the literature [4].

The current study aimed to report the experiences of patients with open pelvic fractures and the treatment protocols. The functional outcomes over an average follow-up duration of 36 months were also examined. Additionally, the etiologies that might be associated with worse functional outcomes were also studied.

## Materials and methods

The data and images of patients with open pelvic fractures visiting our institute between January 2014 and June 2018 were retrospectively collected from the registration database of the institute. Patients with (1) open pelvic fracture (2) osteosynthesis for the pelvic ring injury (3) age ≥ 18 years, and (4) complete radiological and functional follow-ups were included. The patients who did not meet the inclusion criteria were excluded.

The data on age, gender, location of resuscitation, mechanism of injury, associated classifications for the open pelvic fracture, injury severity score (ISS), new injury severity score (NISS), numbers of surgery, follow-up duration, and time to union of the enrolled patients were reviewed and recorded.

### Resuscitation protocol

**Fig. 1:**
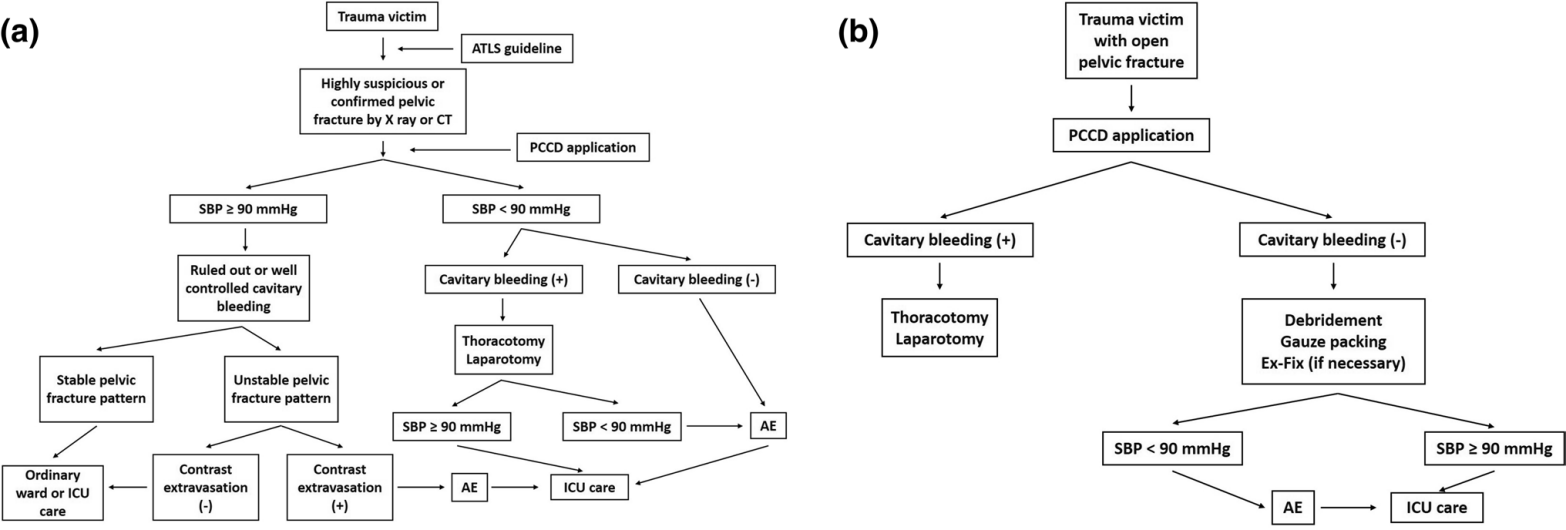
Resuscitation protocol of patients with pelvic fracture. **a** Closed pelvic fracture. **b** Open pelvic fracture

The resuscitative protocols for patients were classified into two categories based on the type of pelvic fracture (closed or open, Fig. 1). For closed pelvic fractures, patients were resuscitated and managed according to our established protocol, which is primarily based on the Advanced Trauma Life Support Guidelines. If these patients developed shock, a blood transfusion with packed red blood cells, fresh frozen plasma, and platelets in a 1:1:1 ratio was provided. Furthermore, when they were unresponsive to fluids and blood resuscitation, arterial embolization (AE) was performed as the priority resuscitation procedure to stop retroperitoneal bleeding, in case of major bleeding originating from the pelvic fracture.

In contrast, in patients with open pelvic fractures, procedures such as surgical debridement, gauze packing of the open surgical wound, and external fixation in cases of pelvic instability were executed following primary resuscitation. The blood transfusion protocol was similar to that for closed pelvic fractures. Life-saving surgeries, such as thoracotomy and laparotomy, were performed simultaneously or sequentially during damage control orthopedic procedures in the operation theater. If the hemodynamic status remained unstable, AE was performed after life-saving procedures to facilitate hemostasis from the pelvic region. The packed gauzes were usually removed at 24 h after the surgery, and repeated debridement procedures were usually performed to decrease contamination. The timing for other surgeries was chosen based on the patient’s condition. Pelvic osteosynthesis was performed as soon as possible after the patient had been hemodynamically stabilized.

While diversional colostomy was not a routine procedure for these patients, it was usually performed within 48 h after the trauma. The decision for diversional colostomy was collectively made by the traumatologist and the orthopedic traumatologist. Diversional colostomy was recommended in two circumstances: (1) for an open wound located at the perineum or the existence of rectal injury referring to Faringer zone I injury [8] and (2) the existence of bowel injury, for which the patient underwent bowel resection/anastomosis surgery, and the procedure of diversional colostomy was performed by the traumatologist.

### Fracture and associated classifications

In addition to patients’ demographic data, several classifications and scoring systems were used in this study. Trauma scores, such as the Injury Severity Score (ISS) and New Injury Severity Score (NISS), which are generally applied to patients after major blunt trauma were used [9, 10].

The Arbeitsgemeinschaft für Osteosynthesefragen (AO) classification for pelvic fracture was applied to determine the stability of pelvic ring injury [11]. This classification divides patients into three groups according to the severity and complexity of their injury: type A, stable pelvic ring injury; type B, partially unstable pelvic ring, and type C, completely unstable pelvic ring.

To represent the impact of open wounds related to pelvic fractures, Faringer and Jones–Powell classifications [8, 12], which focus solely on the location of open wounds and location of open wounds with fracture stability, respectively, were used specifically for open pelvic fractures. The Faringer classification was developed to classify open pelvic fracture by anatomical location of the wound: zone I (perineum, anterior pubis, medial buttock, and posterior sacrum), zone II (medial thigh and groin crease, and zone III (posterolateral buttock, iliac crest). The Jones–Powell is composed of the open wound site and fracture stability and classified into class 1 (stable pelvic ring), 2 (unstable pelvic ring without rectal or perineal wound), and 3 (unstable pelvic ring with rectal and/or perineal wound).

### Radiological evaluation

Radiological outcomes were determined through postoperative radiography using three standard pelvic radiographic views: anteroposterior, inlet, and outlet views. We applied the picture archiving and communication system to adjust the magnification of the area of interest. All radiographs were assessed using a similar method. Regarding the quality of post-osteosynthesis, we adapted the Matta/Tornetta and the Lefaivre criteria [13–15] to evaluate vertical displacement and pelvic symmetry, respectively (Table 1).

**Table 1:** Grading of radiological outcomes based on Matta/Tornetta and Lefaivre criteria [13]

	Matta/Tornetta criteria displacement (mm)	Lefaivre criteria displacement (mm)
Excellent	< 4	< 5
Good	4–10	5–10
Fair	10–20	10–20
Poor	> 20	> 20

All images were primarily reviewed by two surgeons. If the surgeons had similar interpretations, the mean score was recorded. In the cases of different interpretations but similar scores, the differences were overlooked, and the scores were reported. However, if both surgeons provided different interpretations and scores, another senior surgeon (Y.-H, Y.) interpreted the images to determine the final score.

### Rehabilitation protocol and functional outcome evaluations

The rehabilitation protocol for these patients was individualized according to their concomitant injuries. Generally, patients were allowed to perform bedside rehabilitation exercises, such as rolling and passive/active muscular stretch exercises, once their pelvic ring injury had stabilized. When all injuries had stabilized, training for crutch- or wheelchair-assisted ambulation was performed. There was no routine pharmaceutical prophylaxis for venous thromboembolism (VTE). However, VTE was mechanically prevented with the use of compression socks in each patient if there was no contraindication for such therapy (for example, cases such as degloving injury of the lower extremities). Functional outcome evaluations (Merle d’Aubigné score and Majeed hip score) were performed at 3, 6, and 12 months and thereafter annually after discharge from the hospital [16–19].

### Statistical analysis

Statistical analyses were conducted using SPSS version 18.0 (SPSS Inc., Chicago, IL, USA). Continuous variables were reported as means ± standard deviations and medians (ranges). Categorical variables were expressed as numbers (percentages). The cohort in this study was not normally distributed as found by Shapiro–Wilk test. Therefore, the study employed a nonparametric Mann–Whitney *U* test for the analysis of continuous variables. A two-tailed *P*-value of 0.05 was considered statistically significant.

## Results

During the study period, a total of 772 patients with pelvic fractures were primarily resuscitated or transferred to the emergency department (ED). Among these patients, 37 (4.9%) were diagnosed with an open pelvic fracture. Eight patients failed to respond to resuscitation and died at the ED due to multiple injuries. Overall, the mortality rate for patients with pelvic fractures was 21.6%.

After resuscitative procedures, 29 patients survived and were discharged after complete treatment courses. Of these, eight patients who did not undergo osteosynthesis (six patients refused surgical intervention because of the patients’ or the relatives’ consent, and two were too sick to be operated within the recommended time) and two who were lost to follow-up, were excluded. Finally, 19 patients were included in this study. All the 19 patients were operated on and cared by two surgeons (I-C. T. and Y.-H. Y.).

Demographic data of the patients are presented in Table 2. There were 10 male and 9 female individuals with a mean age of 37.5 ± 17.8 years. There were four, six, and nine patients with AO type 61-A, 61-B, and 61-C pelvic ring fractures, respectively. There were 11, 1, and 7 patients with open wounds related to the pelvic fractures located in Faringer zones I, II, and III, respectively. All fractures were united at the mean time of 7.0 ± 2.5 months.

**Table 2:** Demographic data of survivors with open pelvic fractures who completed treatment and follow-up courses

	*N* (%)	Mean (SD)	Median (range)
Sex			
Male	10 (52.6)	NA	NA
Female	9 (47.4)	NA	NA
Age (years)	–	37.5 (17.8)	35 (6–74)
Location of primary resuscitation			
Other hospitals	9 (47.4)	NA	NA
Our institute	10 (52.6)	NA	NA
Mechanism of injury			
Motorbike accident	7 (36.8)	NA	NA
Car accident	4 (21.1)	NA	NA
Fall injury (> 6 m)	3 (15.8)	NA	NA
Pedestrian injury	3 (15.8)	NA	NA
Crushing injury	2 (10.5)	NA	NA
Fracture classification^a^			
61-A	4 (21.1)	NA	NA
61-B	6 (31.2)	NA	NA
61-C	9 (47.4)	NA	NA
Faringer zone			
I	11 (57.9)	NA	NA
II	1 (5)	NA	NA
III	7 (36.8)	NA	NA
Jones–Powell classification			
1	4 (21.1)	NA	NA
2	4 (21.1)	NA	NA
3	11 (57.9)	NA	NA
Diversional colostomy			
No	10 (52.6)	NA	NA
Yes	9 (47.4)	NA	NA
Arterial embolization			
No	11 (57.9)	NA	NA
Yes	8 (42.1)	NA	NA
Treatment protocol			
Single-stage treatment	9 (47.4)	NA	NA
Multi-stage treatments	10 (52.6)	NA	NA
Numbers of surgery	NA	5.5 (3.6)	5 (1–14)
Admission day	NA	25.7 (13.5)	25 (7–67)
ISS	NA	23 (12.1)	20 (9–43)
NISS	NA	29 (12.0)	27 (9–48)
Mean follow-up (months)	NA	39.2 (8.7)	36 (30–63)
Time to union (months)	NA	7.0 (2.5)	6 (4–12)

^a^The classification of pelvic ring injury is based on Arbeitsgemeinschaft für Osteosynthesefragen classification (2018)

*ISS* Injury Severity Score*, NA* not available*, NISS* New Injury Severity Score, *SD* standard deviation

The associations among concomitant injuries and fracture types are shown in Table 3. The more severe fracture types were accompanied by higher number concomitant injuries, such as extremity fractures, urogenital injury, and rectal injury. Thus, the more severe the fracture, the higher the severity scores.

**Table 3:** Associations between concomitant injuries and fracture types of pelvic ring injury

Associated injuries	Arbeitsgemeinschaft für Osteosynthesefragen classification for pelvic ring injury			
	61-A	61-B	61-C	*N*
	A1 (*N* = 2)	B1 (*N* = 1)	C1 (*N* = 5)	
	A2 (*N* = 2)	B2 (*N* = 4)	C2 (*N* = 1)	
		B3 (*N* = 1)	C3 (*N* = 3)	
Head	0	0	0	0
Face	0	1	0	1
Thorax	1	2	3	6
Abdomen	0	3	1	4
Extremity fractures	1	5	6	12
Spine fracture	0	0	2	2
Urogenital injury	0	1	4	5
Rectal injury	0	0	5	5
Faringer zone				
I	0	4	7	11
II	1	0	0	1
III	3	2	2	7
ISS (mean)	13	22	28	NA
NISS (mean)	16	30	32	NA

*CRIF* closed reduction and internal fixation*; Ex-Fix,* external fixator*; IS,* iliosacral screw*; NA,* not available*; ORIF,* open reduction and internal fixation*; TITS,* trans-iliac-trans-sacral screw

Table 4 summarizes the treatment methods according to different injury patterns. Conservative treatment (52.6%) was the most common treatment for anterior pelvic ring injury, followed by percutaneous fixations (36.8%), including anterior columns screws, pubic screws, external fixator, and anterior superficial internal fixator (ASIF), and then by open reduction and internal fixation (ORIF, 10.5%). In contrast, for posterior pelvic ring injury, ORIF (47.4%) was the most common procedure performed using different approaches, followed by percutaneous (42.1%) and conservative (10.5%) treatments. Percutaneous treatment, either with an external fixator or screws, was the most frequent strategy for the loss of fixation injuries before fracture union.

**Table 4:** Summary of approaches for osteosynthesis and related implants

	Patient number, *N* (%)								
		CRIF	ORIF						
	Conservative treatment	Percutaneous (IS, TITS, Ex-Fix)	Lateral window	Dorsal approach	Pfannenstiel	Ilioinguinal	Iliofemoral	Kocher-Langenbeck	Spinopelvic osteosynthesis
Anterior pelvic ring	10 (52.6)	7 (36.8)	0	0	2 (10.5)	0	0	NA	NA
Posterior pelvic ring	2 (10.5)	8 (42.1)	2 (10.5)	2 (10.5)	NA	1 (5)	1 (5)	1 (5)	2 (10.5)
Loss of fixation	NA	3 (15.8)	0	0	0	0	0	0	0

*CRIF* closed reduction and internal fixation; * Ex-Fix* external fixator; *IS* iliosacral screw; *NA* not available;* ORIF* open reduction and internal fixation;* TITS* trans-iliac-trans-sacral screw

**Fig. 2:**
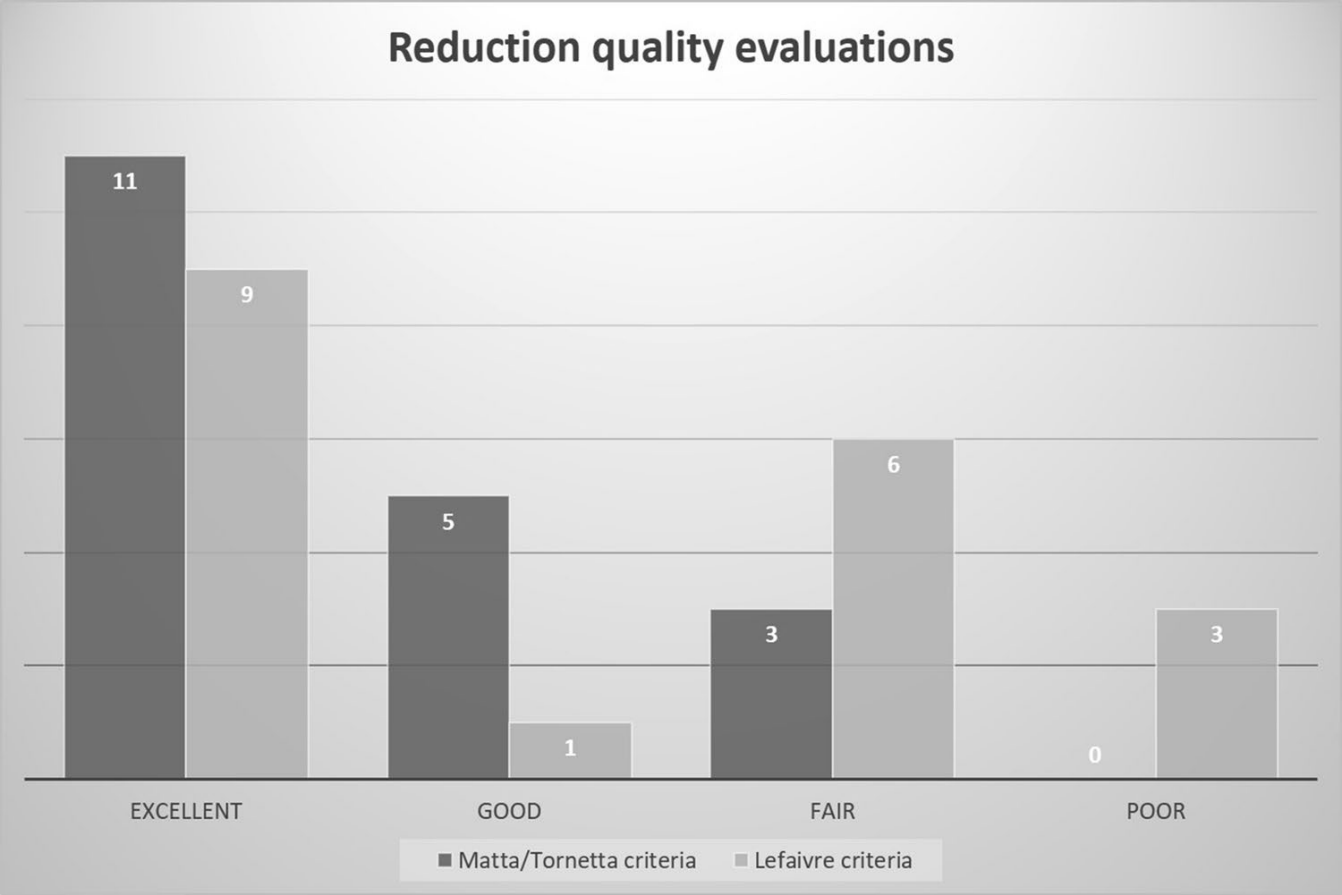
Results of quality of reduction of the pelvic ring by Matta/Torenetta and Lefaivre criteria

**Fig. 3:**
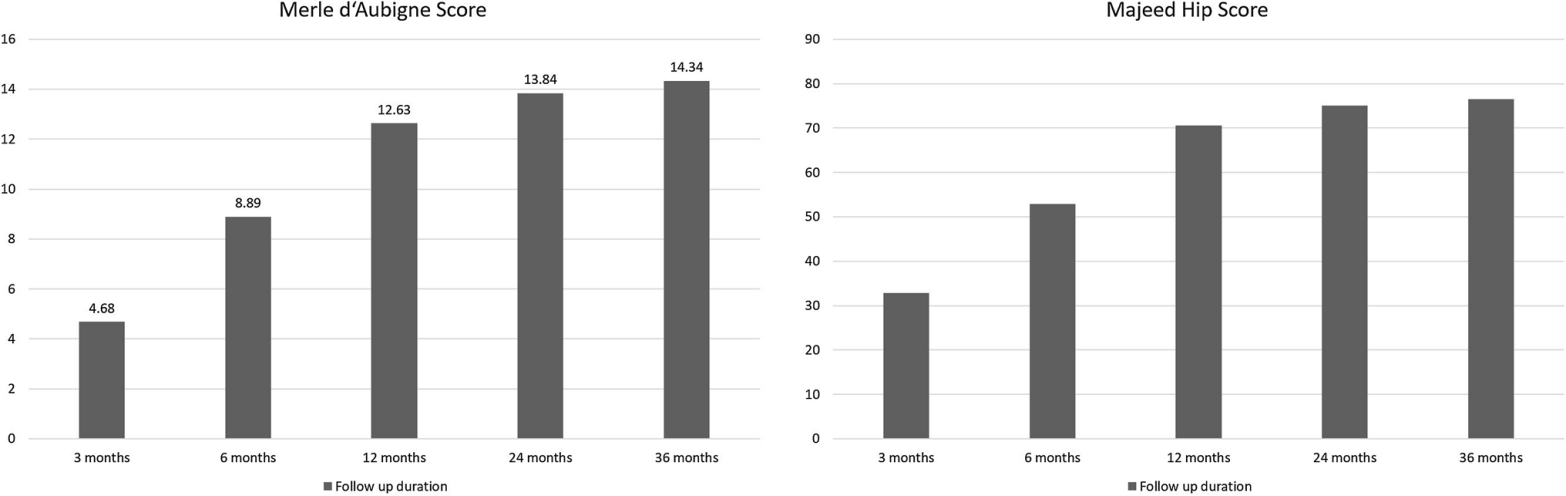
Results of functional outcome evaluations with Merle d’Aubigné score and Majeed hip score. **a** Merle d’Aubigné score. **b** Majeed hip score

All the patients completed the radiological and functional outcome evaluations. As shown in Fig. 2, 11 of 19 (57.9%) and 9 of 19 (47.5%) patients showed excellent reduction quality by Matta/Torenetta and Lefaivre criteria, respectively. Functional evaluations were performed at scheduled intervals (Fig. 3). Statistical analysis revealed that the Merle d’Aubigné score improved significantly at each evaluation. However, the improvement stopped from the 24-month to the 36-month evaluation. A similar trend was found for the Majeed hip score. However, there was no significant difference in improvements when comparing the 12- and 24-month, 12- and 36-month, and 24- and 36-month evaluations.

**Fig. 4:**
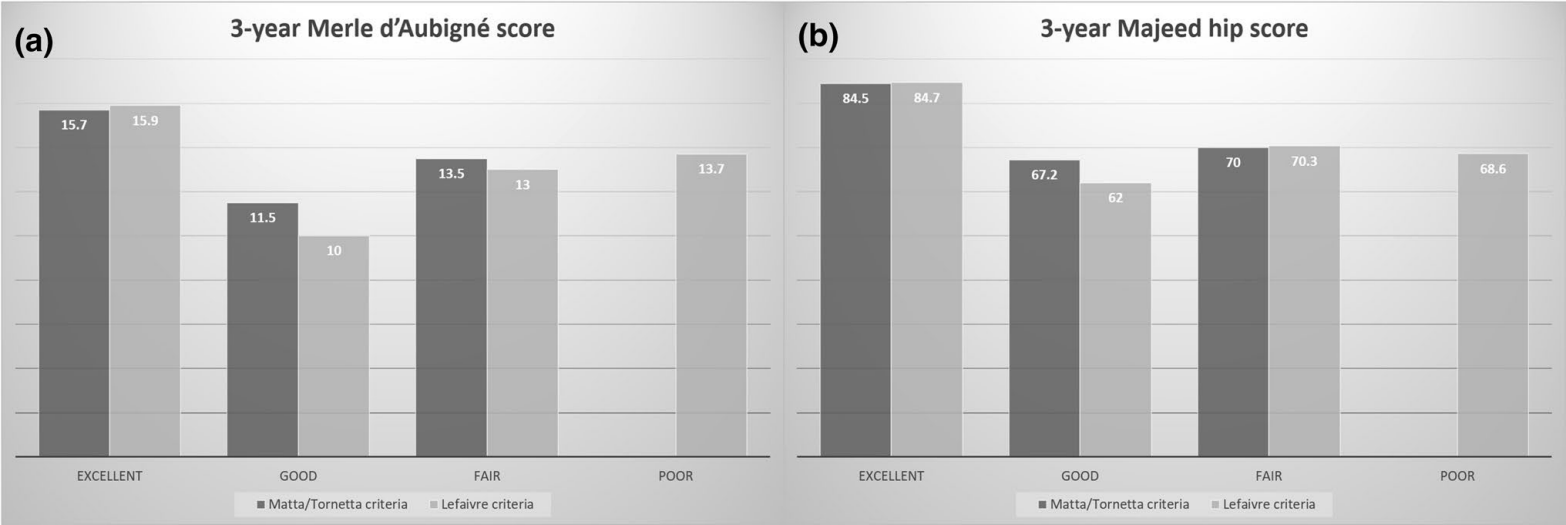
The relationship between the quality of reduction for the pelvic ring and 3-year functional outcomes. **a** Merle d’Aubigné score. **b** Majeed hip score

We further analyzed the relationship between the quality of reduction for the pelvic ring and 3-year functional outcomes (Fig. 4). The patients with initial excellent reduction quality of the pelvic ring showed the highest score in both score systems. However, patients with good reduction quality of the pelvic ring (*n* = 2) showed the lowest scores either by Matta/Torenetta or Lefaivre criteria. In addition to open pelvic ring injury, lumbar spine and sacral fractures with lumbosacral plexopathy were also observed, and the symptoms of lumbosacral plexopathy hardly improved, even 3 years after osteosynthesis.

## Discussion

The optimal treatment protocol for pelvic fractures remains debatable. The resuscitation protocol for such injuries should be based on the facilities available at the medical institute, and the aim should be to perform life-saving procedures without causing additional harm to the patient. In this study, 8 of 37 patients with open pelvic fractures died during resuscitation at the ED. Injuries to vital organs, such as the brain, chest cavity, or abdominal cavity, or the open pelvic fracture itself, were the causes of death in these patients. Those who were successfully resuscitated survived the treatment protocol and received complete treatment. Under the protocol for selective diversional colostomies, scheduled debridement surgeries, tailor-made design of osteosynthesis, and individualized rehabilitation protocols, the patients were expected to return to their daily activities, even when the initial functional status was poor.

Open pelvic fracture is a problematical injury not only because of the high mortality rate during the trauma but also due to high infection rates during the treatment [1, 20–22]. The colonized bacteria may originate from contamination by the environment or more likely due to the presence of fecal content in the retroperitoneal cavity. Faringer et al. [8] reported the infection rates in relation to the location of open wound and classified them into zones I, II, and III. They further advocated that all zone I open pelvic fractures should undergo diverting colostomy. Some reports support the statement that diverting colostomy is indicated only for patients with rectal injury despite an open wound that is present in zone I [10, 23, 24]. In our study, there were nine patients (eight with zone I injury and one with zone III injury) who underwent diversional colostomy. The aim of our protocol was to prevent potentially infective sources from reaching the pelvis. The fact is that patients with pelvic fractures might be bed-ridden for a prolonged period, and the wound may get contaminated by fecal matter under this circumstance. For this reason, we believe that fecal contamination, which may lead to infection and sepsis, might be avoided by promptly performing diverting colostomy. Under this protocol, no patient experienced sepsis during hospital stay or chronic osteomyelitis after discharge.

The best surgical strategy to perform osteosynthesis for pelvic fractures remains controversial. The management of the anterior pelvic ring has evolved with the introduction of various types of external fixators, intramedullary implant fixation, plate and screw, and ASIF [25–28]. Although various approaches could be used, only two patients received ORIF with plate and screw because of disrupted pubic symphysis through Pfannenstiel incision. In fractures with main wounds located anteriorly, we tried to provide a less aggressive treatment to avoid possible surgical contamination. In contrast, for posterior pelvic ring injury, the treatment policy was more aggressive. Open reduction through various approaches, as mentioned above, aimed to restore the anatomy and stability of the posterior pelvic ring for the purpose of lessening sequelae from prolonged immobilization and malunion. Similar concepts were mentioned by the previous reports for closed pelvic injuries [4, 29, 30]. We believe that although the possibility of surgical site infection following osteosynthesis for posterior pelvic ring injury exists, adequate stability of the posterior pelvis is still crucial in such patients for early mobilization. As a result of employing a protocol for early mobilization, three cases showed loosening of implants, one with ASIF and two with percutaneous iliosacral screws.

Several possible complications may occur after pelvic fractures, such as gait disturbance, chronic pelvic pain, and functional disability [31, 32]. These complications may be the sum of the complexity of fracture types, surgical approach, and concomitant injuries. Especially in open pelvic fractures, necessary life-saving procedures, such as TAE, limb amputation, and diversional colostomy, may be the cause of temporary functional deficits [25, 33, 34]. Kokubo et al. [7] reported that lower extremity fractures, conservative therapy, and nerve damage affected the 1-year functional outcomes. Additionally, prolonged time for osteosynthesis also led to worse long-term functional outcomes [33]. Among the enrolled patients, fractures in 15 patients were classified as Jones–Powell 2 or 3, with the ISS being 22.5 for AO type B and 28.1 for AO type C. We observed that the functional evaluations during the first 3 months were poor. Although long-term evaluations are lacking, the patients’ functions improved initially with each evaluation, but stagnated after 24 months. Additionally, despite good reduction quality evaluated by applied criteria in two patients, they revealed unsatisfactory functional outcomes 36 months after osteosynthesis because of persistent leg numbness, pain, and weakness which are the sequelae of lumbosacral plexopathy after lumbar and sacral fractures. Therefore, we postulated that permanent injuries from severe trauma might have contributed to the stagnation in patients’ satisfaction.

Despite our efforts to avoid bias during data collection and analysis, some limitations exist in this study. First, only 19 patients were enrolled in this study. Thus, this cohort is relatively small. However, the incidence of open pelvic fractures is reportedly low, from 2 to 4% of all pelvic fractures [4], including our study. The considerable rate of mortality among our patients made the enrolled numbers low. Second, although there was a uniform treatment protocol and rehabilitation program for the patients with open pelvic fractures, the locations of the open wound, concomitant injuries, and complexity of fractures were not similar. This diversity among patients’ factors might have interfered with the study results. Finally, only two functional evaluations were conducted to evaluate the functional status after trauma and osteosynthesis. Further pivotal clinical studies will benefit from the application of different evaluation tools, such as 36-item short form survey and sexual function assessments, to demonstrate the real functional status of these patients.

## Conclusions

Although the mortality rate in this study was considerable, patients had a good chance of receiving a complete treatment course if they survived the resuscitation. The approaches for the treatment of fractures should be individualized according to the fracture pattern, location of the open wound in the pelvis, and concomitant injuries. Despite the poor initial functional scores, functional improvements may be anticipated. Additionally, an anatomical restoration of the pelvic ring suggested a better functional performance at least a 36-month follow-up. A further study should be conducted to follow these patients in order to obtain long-term functional outcomes.

## Data Availability

All data generated or analyzed during this study are included in this published article. The datasets used and/or analyzed during the current study are available from the corresponding author on reasonable request.
